# The Role of Electronic Cigarettes in Dental Caries: A Scoping Review

**DOI:** 10.1155/2023/9980011

**Published:** 2023-08-30

**Authors:** Sumit Gaur, Rupali Agnihotri

**Affiliations:** ^1^Department of Pedodontics and Preventive Dentistry, Manipal College of Dental Sciences, Manipal Academy of Higher Education (MAHE), Manipal, Karnataka 576104, India; ^2^Department of Periodontology, Manipal College of Dental Sciences, Manipal Academy of Higher Education (MAHE), Manipal, Karnataka 576104, India

## Abstract

Dental caries, a predominant childhood and adolescence affliction, has numerous factors implicated in its pathogenesis. Electronic cigarettes (ECs) have recently gained popularity among the younger population. Various factors, such as the EC liquid composition and aerosols, are associated with the development of dental caries. This review explains numerous EC-related factors which may lead to dental caries. An electronic search was conducted in Medline (Pubmed), Scopus, and Embase databases to evaluate the original research investigating the role of ECs in developing dental caries. About 12 included studies in the review indicated that factors such as the composition of e-liquids and aerosols are significant in the progression of dental caries. Specifically, cariogenic sugars such as sucrose, fructose, glucose, aldehydes, and flavors such as menthol, cinnamon, and strawberry in the e-liquids contribute to dental caries. They are toxic to oral commensals such as *Streptococcus gordonii*, *Streptococcus mitis*, *Streptococcus intermedius*, and *Streptococcus oralis* and promote the proliferation of cariogenic pathogens such as *Streptococcus mutans* (*S. mutans*) which causes dental caries. However, further validation of the effects of ECs on the development of dental caries is warranted through clinical trials.

## 1. Introduction

About 3.5 billion people worldwide are affected by oral diseases, of which approximately 2 billion people suffer from caries of permanent teeth, while 520 million children suffer from primary teeth caries [1]. The global rise in dental caries is attributed to increased urbanization and changes in living conditions. Lately, foods and beverages with high sugar, tobacco, and alcohol have become popular among youngsters and adolescents, resulting in poor oral health conditions such as dental caries [1].

China patented electronic cigarettes (ECs) in 2003 and launched them in the European and North American markets in 2006 [2]. They were sold as an economical and safer substitute for conventional cigarettes (CCs) and a smoking cessation method [3]. Lately, a more significant number of young adults are experimenting with ECs than aged individuals. Approximately, 5% of middle and 20% of high schoolers used them within 30 days in 2020 [3]. The Truth Initiative reported that 32.7% of high school students aged 14–18 practiced vaping, which heightened the risk of a tobacco epidemic [3]. It is deplorable that vaping, initially introduced as a method for smoking cessation, exposes our younger generation to various addictives such as nicotine.

ECs are devices in which liquids are heated to form a dense aerosol inhaled by the user [3]. They comprise a mouthpiece, an e-liquid tank, a heating element, and a battery. They are available in different forms, shapes, colors, and flavors. The aerosol is absorbed into the bloodstream or adheres to the oral cavity structures [4]. The exposure to nicotine depends upon the composition of the liquid, usage, and factors related to the device. More than 400 brands and 7000 unique flavors of e-liquids are commercially available [5, 6]. They are plausibly responsible for the adverse oral and systemic health effects as they contain aldehydes and free radicals leading to oxidative stress, DNA damage, altered antioxidant activity, and protein carbonylation [7–9]. The ECs caused 2,807 lung injuries and more than 52 deaths, as reported in the 2019-2020 Centres for Disease Control and Prevention report [7, 10].

Sweet substances such as propylene glycol and glycerin in the e-liquid base dissociate into by-products such as acetic acid, lactic acid, and propionaldehyde, intensifying enamel demineralization [3, 7]. Furthermore, EC aerosol and nicotine promote xerostomia and increase the attachment of *Streptococcus mutans* (*S. mutans*) on the enamel surface [4, 7]. Some e-liquids contain increased cariogenic sugars such as fructose and sucrose [4, 6, 11]. These factors create a favorable environment for the progression of dental caries, specifically pit and fissure caries [7]. With this background, this review aims to gain insight into the cariogenic potential of ECs.

## 2. Methods

**Figure 1:**
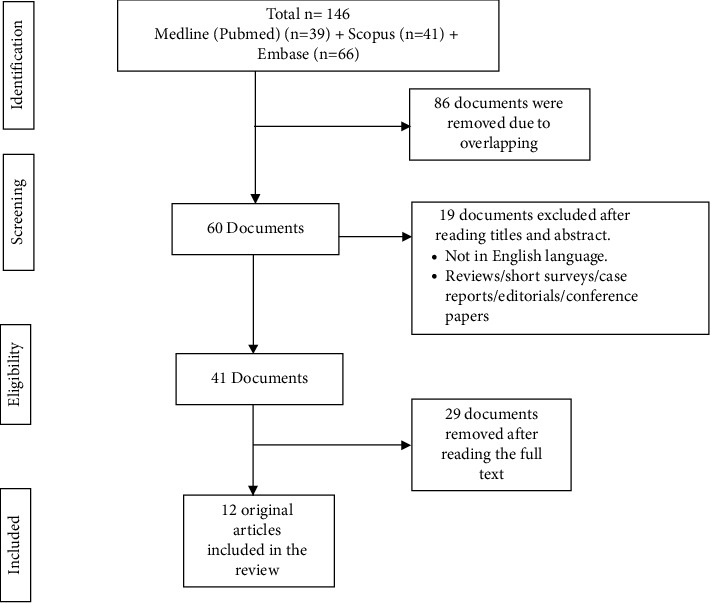
Evidence search for the role of electronic cigarettes in dental caries.

This review was conducted according to the Preferred Reporting Items for Systematic reviews and Meta-Analyses extension for Scoping Reviews (PRISMA-ScR) guidelines [12]. The research question was what is the role of ECs in developing dental caries? The articles related to the cariogenic effects of ECs were identified from the databases such as Medline (PubMed), Scopus, and Embase till 13th July 2022. During the initial search, a keyword combination including “Dental Caries” AND “Electronic Cigarettes” OR “e-cigarette” OR “Electronic nicotine delivery system” OR “Electronic nicotine delivery device” OR “e-liquid” OR “Vaping” was confirmed in the titles, abstracts, or keywords resulting in 146 articles (Figure 1). The titles and abstracts of 60 articles were read. Only original studies in English related to the cariogenic effects of ECs were included. Any recommendations, animal studies, conference proceedings, expert statements, reviews, technical reports, and nonoriginal papers were excluded. Finally, 12 original studies were included, and the data extracted were the study type, aims and objectives, composition of the e-liquid, their role in developing dental caries, results, and conclusions [3, 4, 6, 7, 13–20] (Table 1).

## 3. Results and Discussion

### 3.1. Types of Studies

The studies included were either case-control [14, 16] or cross-sectional [7, 15] and in vitro studies [3, 4, 6, 13, 17–20]. Some studies compared clinical oral health parameters between nonsmokers (NSs), CC, and EC users [14–16].

### 3.2. Studies Reporting the Association between Dental Caries and ECs

The prevalence of vaping was high in youngsters aged 18 to 24 [7, 14, 16] with a more significant number of male users [16]. It was about 2.75 times the overall prevalence in the study population, including 4,618 participants, of which 247 were EC users [7]. The current EC and the dual (using both ECs and CCs) smokers were 25 to 64 years old, non-Hispanic Blacks, lower than high school education, low socioeconomic status, and infrequent dental visitors (>6 months) and were more susceptible to untreated caries [7]. Poor oral health was reported in daily and intermittent EC users [15, 21–24]. Contrarily, the association between dental caries and EC usage was not significant in another study [14, 16].

### 3.3. Type of E-Liquids

The in vitro studies utilized commercially available e-liquids [6, 17, 19, 20] or prepared them in the laboratory [3, 4, 18]. The sample e-liquids contained propylene glycol and vegetable glycerine at 50 : 50 [3, 17, 18, 20] or 70 : 30 [19] or 20 : 80 [4] ratio with or without nicotine and flavorings, discussed in a later section.

### 3.4. In Vitro Simulation of EC Aerosol Generation

In in vitro studies, vaping was simulated by aerosolizing e-liquids by heating with a fourth-generation EC (G-Priv Baby kit) with one 10-second puff equivalent to a 5-minute exposure [20]. The Tripl3 eGo style lithium-ion battery [17, 18] or eGo ONE CT [19] were used in other studies with different protocols equivalent to 100 [18] or 0, 2, 5, 10, 25, 50, and 75 puff cycles [17] or two puffs every 60 seconds with a 4- to 5-second puff followed by a 25–30 seconds pause [19]. A study also utilized a universal electronic cigarette testing machine for generating EC aerosol wherein ten puffs and 150 puffs simulated a single vaping session or single day use, respectively [4].

### 3.5. Factors Responsible for Cariogenic Effects of ECs

It was evident from the results of the included studies that the sugars and flavors in e-liquids heightened the risk for dental caries. The users orally inhaled the e-liquid aerosol generated upon heating, promoting dental caries through various mechanisms.

**Figure 2:**
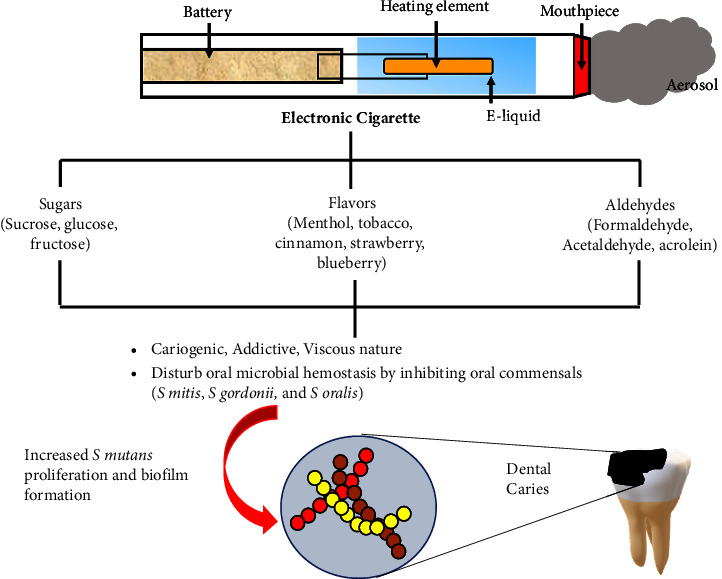
Parts of an electronic cigarette and its role in dental caries.

These factors implicated in the cariogenic effects of ECs are explained as follows (Figure 2).

## 4. Sugars in E-Liquids

In general, added sugars enhance the flavor of tobacco products through sensory modification [6]. Besides imparting a sweet taste, they alter the bitterness of nicotine, reduce its harshness and irritation, and improve the tobacco smoke aroma [6, 25]. The monosaccharides (e.g., glucose and fructose) and disaccharides (e.g., sucrose) either occur naturally in tobacco plants or form during their priming and curing [6]. About 40 to 50% of tobacco products comprise added natural or synthetic sugars that undergo pyrolysis [6].

Besides being cariogenic, sugars in tobacco products modify the sensory impact of tobacco alkaloids and nicotine. They promote opioid and dopamine release, which cause brain stimulation similar to cocaine and morphine [6, 26]. Eventually, these products become more appealing. They activate the Reward Deficiency Syndrome and cause genetic and epigenetic impairment of the brain leading to a craving behavior [26].

Sugars such as glucose (6.4–88.9 *μ*g/mL), fructose (8.8–331.2 *μ*g/mL), and sucrose (9.3–620.1 *μ*g/mL) were detected in the commercially available e-liquids. Glucose exceeded the limits of quantification in about 22% of the samples, while fructose and sucrose in 53% of the samples [6]. Although the sugar levels were not significantly different between the labeled nicotine and zero nicotine e-liquids, the sucrose levels were significantly higher than glucose and fructose in flavored e-liquids. For instance, flavors with 11 mg/mL of nicotine had 9.3 *μ*g/mL of sucrose, 18 mg/ml of nicotine had 88.9 *μ*g/mL of glucose, and 24 mg/mL of nicotine had 8.8 *μ*g/mL of fructose, while flavors with zero nicotine had 6.4 *μ*g/mL of glucose, 331.2 *μ*g/mL of fructose, and 497.0 *μ*g/mL sucrose [6].

Sucrose was also identified in 37 samples of popular e-liquids, but no direct relationship was observed between its levels and the flavor, which varied between different manufacturers. For instance, the highest levels in *μ*g/g of sucrose ranged from 11.67 to 23.73 for menthol, 7.315 to 72.93 for chocolate, 20.15 for Marlboro, 3.40 for coffee, and 29.82 for tobacco flavors, while the lowest levels of sucrose in *μ*g/g were 1.11 for Black, 0.68 to 1.211 for Camel, 0.784 for L & M, 0.62 for Cherry, 0.76 for coffee, and 1.80 for fruit mix flavors [13].

Therefore, the levels of cariogenic sugars such as sucrose were significantly higher in the commercially available e-liquids in the studies [6]. However, the cariogenic effects of inhaled sugars via ECs need future verification.

## 5. Aldehydes in E-Liquids

Usually, various ratios of propylene glycol and glycerine in e-liquids form harmful aldehydes such as formaldehyde, acetaldehyde, and acrolein when heated [27]. Their levels vary with the temperature, type of sugar, tobacco, and other constituents. As the boiling temperature of propylene glycol is 188°C and the working temperature of ECs is around 250°C, aldehydes and organic acids may form even at temperatures of 200°C [13].

In the included studies, aldehyde levels varied between the brands, flavors, and nicotine concentrations. They included formaldehyde (14–368 ng/mL), acetaldehyde (2.1–4676.1 ng/mL), acrolein (0.3–10.1 ng/mL), and vanillin (96.6–11936.2 *μ*g/mL). The levels of formaldehyde and acrolein were significantly associated with fructose and sucrose levels [6].

As acetaldehyde has addictive properties, it may promote nicotine self-administration, while formaldehyde, acrolein, and vanillin in EC aerosols may produce adverse health effects [6, 28]. As some ECs allow the user-controlled modification of battery voltage, increased heat generation from the devices may result in higher levels of aldehydes in the aerosol and increased exposure to harmful constituents [6, 29].

## 6. Flavors in E-Liquids

### 6.1. Types of Flavors

The most common flavors tested were menthol, tobacco, cinnamon, strawberry, and blueberry, which are popular among the youth [3, 18–20]. These flavorings were added in concentrations ranging from 0 to 25% [3, 18]. Besides synthetic sweet flavors such as ethyl butyrate (11.1 mg/mL), ethyl maltol (27.2 mg/mL), hexyl acetate (2.5 mg/mL), sucralose (2.0 mg/mL), and triacetin (11.6 mg/mL) were evaluated [4]. They are esters, sugar alcohol, and sugar substitutes that mimic the taste or smell of flavors such as pineapple (11.1 mg/ml), cotton candy (27.1 mg/ml), apple (2.5 mg/ml), sweetener (2 mg/ml), and velvety smoke (11.6 mg/ml) [4]. A study also analysed 16 commercially available flavors of e-liquids across eight brands [6].

### 6.2. Influence of Flavors on Oral Commensals and *S. mutans* Growth

The 100% concentration of flavors such as menthol, cinnamon, and strawberry in e-liquids was toxic to oral commensals such as *S. gordonii*, *S. mitis*, *S. intermedius*, and *S. oralis* compared to the flavorless e-liquids. They impaired bacterial growth at high concentrations (5 to 25%) in a dose-dependent manner [3, 18]. Various concentrations of humectants or nicotine in homemade flavored e-liquids reduced or augmented their growth [3].

The menthol and cinnamon inhibited *S. intermedius*, while cinnamon, strawberry, blueberry, and menthol affected *S. mitis*, *S. gordonii*, and *S. oralis* [18]. 5% concentrations of cinnamon and menthol inhibited all the streptococci, while 3% cinnamon produced the lowest biofilm mass of *S. intermedius*, lower than that produced by tobacco, strawberry, and blueberry flavors.

Interestingly, at 1% concentration, these flavors affected the biofilm mass, similar to the positive control. The cinnamon and menthol flavors inhibited both single and multispecies biofilm formation and growth in a dose-dependent manner. The tobacco flavor reduced the number of colonies, while the menthol, cinnamon, strawberry, and blueberry completely obliterated the growth. As cinnamon and menthol flavors strongly inhibited biofilm formation, and tobacco flavor only reduced the number of bacteria, the latter had the most negligible effect on the growth of microbial colonies [3, 18].

There was no difference in the single-species biofilms grown in strawberry-flavored and flavorless e-liquids. However, the multispecies biofilm biomass reduced significantly, indicating increased sensitivity of microbial communities to strawberry flavor [3]. The flavorings had no bacteriolytic effects, but they reduced the number of viable bacteria, specifically *S oralis* [3].

Consequently, these flavoring agents disturbed the oral microbiome hemostasis and promoted caries [3, 18]. Their high and low-level exposure to flavored e-liquids and aerosols affected the multispecies balance in oral biofilms leading to bacterial dysbiosis and caries [3, 18].

### 6.3. Influence of Synthetic Flavors on Oral Commensals and *S. mutans* Growth

Certain synthetic flavors in e-liquids such as ethyl butyrate, triacetin, and hexyl acetate and their by-products, which are esters, create a conducive environment for the growth of *S. mutans* [4]. For instance, oral bacteria such as *Streptococcus salivarius* and *Lactococcus lactis* produce ethyl butyrate, which has a strong pineapple scent [4]. Other bacteria such as S*treptococcus*, *Actinomyces*, and *Lactobacillus* metabolize carbohydrates to acetate in oral biofilm [4]. As *S. mutans* is already exposed to ethyl butyrate and acetate in the oral cavity, it thrives easily in these environments [4]. Similarly, triacetin is an ester, and *S. mutans* possess esterase activity and degrade monomers in dental restorative materials [4, 30]. Therefore, the esters in e-liquid flavors may be its additional food source to flourish in the oral environment. Contrarily, the ethyl maltol in e-liquids with a candy-like fragrance suppresses the *S. mutans* by interfering with its cell membrane integrity [4]. It decreased biofilm development significantly compared to flavorless e-liquids.

Similarly, propylene glycol in e-liquids reduced the overall biomass accumulation due to its bactericidal effect, but *S. mutans* was resistant [20]. Furthermore, these flavors increased enamel demineralization and decreased tooth hardness. About six hours of incubation of *S. mutans* with these flavorings caused hardness loss which was highest with triacetin (27.4 ± 7.1), followed by hexyl acetate (21.5 ± 5.7), ethyl butyrate (15.4 ± 4.0), and sucralose (8.6 ± 5.8) [4].

### 6.4. Influence of Nicotine in E-Liquids on Cariogenic Bacteria

Various nicotine concentrations in the e-liquids were 3 mg/ml [20], 10 mg/ml [4], 18 mg/ml [19], and 20 mg/ml [3, 17, 18]. Other nicotine concentrations evaluated in a single study were 0, 6, 12, 18, and 24 mg/ml [6]. The nicotine-rich ECs significantly increased the *S. mutans* biofilm mass (47 ± 5 mg) than the nicotine-free ECs, following six exposures [19]. It was suggested that nicotine supports *S. mutans* colonization by increasing its viability and biofilm formation. At a 1 to 4 mg/ml concentration, it upregulates its virulence receptor proteins and extracellular polysaccharides expression and stimulates the glycolytic pathway intermediates.

## 7. Effects of EC Aerosol

### 7.1. Influence on Oral Commensals and *S. mutans* Biofilm

Various in vitro studies evaluated the influence of EC aerosols on oral commensals and the opportunistic cariogenic bacterium, the *S. mutans* [4, 19, 20]. Similar to the flavored e-liquids, the EC aerosols dysregulate oral bacterial homeostasis by suppressing the growth of oral commensals and enhancing *S. mutans* biofilm formation. The latter dominates other species due to environmental changes such as density, nutritional availability, and pH that promote tooth adherence and biofilm formation [4, 19, 20]. Unlike *S. sanguinis* and *S. gordonii*, the *S. mutans* was unaffected by menthol flavorings or nicotine in the EC aerosols, leading to increased colonization [20]. A 15 min twice daily exposure to EC aerosol or 10 to 150 puffs increased the adhesive force between the *S. mutans* and enamel surface [4, 19]. Its levels increased in pits, fissures, and smooth surfaces exposed to aerosol after 24 hours of incubation [4]. As the EC aerosols are viscous and cover the enamel surface, they alter the surface interactions of *S. mutans* [4]. It attaches to the exposed surfaces and metabolizes the e-liquid bases and flavors to secrete extracellular polymeric substances that promote encapsulation and multiplication to form biofilm [4]. It rapidly metabolizes the carbohydrates into lactic acid, creating a locally low pH, and demineralizes the enamel surface and dental caries. The adhesive forces between *S. mutans* and the enamel surface depend on the number of puffs. It was shown that the aerosol droplets were evenly distributed in the presence of ten puffs, but as the number of puffs increased, they started aggregating. For instance, aerosols from 0, 10, and 150 puffs deposited 5.7 ± 5.0, 175.5 ± 12.7, and 1051.25 ± 59.4 particles/mm^2^, respectively, which affected bacterial adhesion.

Furthermore, the aerosolization process alters the chemical nature of e-liquids and significantly influences bacterial growth. The heat generated from the atomizer coil generated harmful and toxic vape by-products such as formaldehyde and acetaldehyde from propylene glycol and glycerol, alcohols, aromatic hydrocarbons, carboxylic acids, esters, aldehydes, urea, carbonyl compounds, and others from the flavors when heated [20]. The ethyl maltol produced the maximum by-products, while sucralose produced the least [4]. The ECs also produced toxic metals such as calcium, copper, iron, magnesium, silicon, cadmium, cobalt, chromium, nickel, and palladium from the heating coil and other metal components [4]. These metals can be toxic to bacteria at high concentrations but serve as nutrients at physiological levels. Oral bacteria, including *S. mutans*, require metal ions (e.g., copper, iron, and magnesium) as cofactor to activate essential enzymes [4]. Magnesium is a nutrient source for *S. mutans*. Metals such as calcium, iron, and copper in EC aerosol may modulate biofilm formation and enamel remineralization/demineralization processes [4]. However, contrary effects were reported where the bacteria exposed to flavored EC aerosols grew slower during the exponential phase than those exposed to the e-liquids only [18].

Similarly, the planktonic eight-hour streptococcal growth was unaffected by flavorless EC aerosol (with or without nicotine) and CC smoke although the latter was more detrimental [17]. A comparison of CC smoke with EC aerosols showed that CCs were far more detrimental to the survival and growth of the commensals although nicotine-rich ECs produced effects similar to CC smoke. The *S. mutans* adhered and grew better on teeth previously exposed to nicotine-rich EC aerosol, as nicotine promotes biofilm formation.

### 7.2. Influence on Bacterial Genes

The EC aerosols increase the abundance of bacterial genes encoding quorum sensing, biofilm formation, stress response, and virulence factors [20]. They specifically regulate the expression of biofilm-associated genes such as competence (com) *C*, *D*, and *E*; glucosyltransferase (gtf) *B*, *C*, and *D*; and glucan binding proteins (gbp) *B* and *C* in *S. mutans*. These genes are related to quorum sensing and are essential for cell viability in response to environmental conditions [19]. Exposure to EC aerosol, a stressful condition for *S. mutans*, activates the specific com genes that promote biofilm formation for its protection. However, high levels of mRNA expression of gtf*BCD* and gbp*BC* genes enhanced the expression of the virulence factors from *S. mutans*. It was suggested that e-liquid-associated factors, such as nicotine concentration, flavors, and liquid viscosity, controlled the activation of these regulating (com *C* and *D*) and virulence (gtf*BCD* and gbp*BC*) genes [18]. Subsequently, the number and density of *S. mutans* significantly increased on teeth surfaces exposed to nicotine-rich ECs and CC smoke with simultaneous expression of com *C*, com *D*, gft, and gbp genes, while the expression of com E genes was unchanged [19].

### 7.3. Influence on Bacterial Hydrophobicity

The *S. mutans*, the cariogenic pathogen, possess cell surfaces hydrophobicity features such as hydrophobic amino acid residues, outer membrane proteins, lipids, and lipoteichoic acid. It changes the membrane phenotypes between hydrophilic and hydrophobic during environmental changes such as exposure to EC aerosol, which usually reduces the hydrophobicity of *S. sanguinis* and *S. gordonii*. However, *S. mutans* has higher hydrophobicity than the commensals and shows greater coaggregation and attachment to the OKF6 cells [20]. Besides, it stimulates the expression of IL-8 and antimicrobials so that it is not recognized as pathogenic by epithelial cells and evades the immune response.

### 7.4. Influence of Flavors in EC Aerosol on Host Immune Response

The classic tobacco, cinnamon, and strawberry flavors in aerosols altered the host immune response, influencing microbial growth [18]. They stimulated interleukins (ILs) 1, 6, 8, and 10 and upregulated chemokines such as CXCL1, CXCL2, and CXCL10 [18, 31, 32]. They caused morphological changes in human lung epithelial and fibroblast cells. For instance, cinnamaldehyde, the primary component of cinnamon flavor, decreased the viability of human monocytes and upregulated IL-8 dose-dependently [33]. They impaired the redox balance by suppressing glutathione and glutathione disulphide in the aerosol-exposed tissues [30]. However, menthol and cinnamaldehyde are antimicrobial [18]. They serve as a carbon source at low concentrations and promote bacterial metabolism and growth. In contrast, at high concentrations, their action is similar to antibiotics suppressing the growth of oral commensals. They even supported *S. mutans* biofilm growth in nicotine-free EC aerosols [20].

Even though the included studies show that ECs have cariogenic potential, most of the factors responsible for dental caries were identified in in vitro studies and need future verification through in vivo studies.

## 8. Conclusion

It is evident from the results of the included studies that electronic cigarettes are cariogenic owing to high levels of sugars and flavors in their e-liquids. Heating the e-liquid generates an aerosol that contains numerous harmful by-products such as aldehydes. They disturb the oral microbiome homeostasis leading to bacterial dysbiosis and disease. Moreover, sugars and aldehydes are addictive, which may increase their usage among youngsters. In the future, longitudinal in vivo studies should be performed to implicate their definitive role in dental caries.

## Figures and Tables

**Table 1:** Role of ECs in the development of dental caries.

Author name	Type of study	Salient features of the study	Factor evaluated	Results and conclusion
Kubica et al. 2014 [13]	In vitro	Developed an analytical method for rapidly verifying sucrose and other saccharides in EC e-liquids and evaluated the sugar content in branded e-liquids	Sugars	(i) Sucrose is present in branded e-liquids

Ghazali et al. 2018 [14]	In vivo observational (case-control) study	Evaluated oral health of 120 participants, of which 40 were EC users	EC use	EC users:
				(i) Had increased gingival bleeding
				(ii) DMFT similar to controls
				(iii) Relatively young

Fagan et al. 2018 [6]	In vitro	Quantified sugar levels and aldehydes in branded EC liquids, flavors, and nicotine concentrations	Sugars	Increased:
				(i) Levels of cariogenic sugars such as glucose, fructose, and sucrose, with sucrose levels higher than fructose and glucose
			Flavors	(ii) Aldehydes such as formaldehyde, acetaldehyde, and acrolein, with acetaldehyde being the highest
			Nicotine	(iii) Association of formaldehyde and acrolein with fructose and sucrose
				(iv) Sugars and aldehydes in unheated EC liquids may promote experimentation among youngsters

Huilgol et al. 2018 [15]	In vivo	Evaluated the EC use and oral health link	Frequency of EC use	(i) Daily use of ECs was independently correlated to poor oral health

Ghazali et al. 2019 [16]	In vivo observational (case-control) study	Compared the caries occurrence between the noncigarette and non-EC users, cigarette users, and EC users	EC use	(i) No significant difference in the mean DMFT values between all groups at baseline and 6 months

Nelson et al. 2019 [17]	In vitro	Evaluated the flavorless EC aerosol influence on the planktonic growth of oral commensal such as streptococci and compared the outcomes with those of CC smoke	EC aerosol	(i) E-liquid and aerosol sparsely affected streptococcal growth, while CC smoke hindered streptococcal growth
				(ii) Smoke-treated growth media was more detrimental to oral commensal streptococci than e-liquid or EC aerosol

Kim et al. 2020 [4]	In vitro	Evaluated the cariogenic potential of EC aerosols generated from e-liquids with sweet flavors	EC aerosol flavor	(i) EC aerosols enhanced microbial adhesion to enamel, specifically the *S. mutans*, due to their viscosity, doubled biofilm formation, and reduced enamel hardness
				(ii) Esters in e-liquid promoted enamel demineralization
				(iii) Sugar alcohols inhibited *S mutans* growth and adhesion
				(iv) The EC aerosol's physiochemical properties were similar to high-sucrose, gelatinous candies, and acidic drinks. Viscous e-liquids, together with the chemicals in sweet flavors, increased the cariogenic potential of EC's

Fischman et al. 2020 [18]	In vitro	Tested the influence of popular e-liquid flavorings on the planktonic growth of oral commensals such as streptococci	E-liquid flavor	Flavored E-liquids:
				(i) More unfavorable for oral commensal bacterial growth than unflavored e-liquids
				(ii) Disturb the composition and growth of primary colonizers hindering healthy dental plaque and host-bacteria interactions
				(iii) Alterations in the pioneering oral communities may be harmful to oral health

Rouabhia and Semlali 2021 [19]	In vitro	Evaluated the effects of ECs on *S. mutans* growth, biofilm formation, and virulence genes expression	EC aerosol	(i) Promoted *S. mutans* growth at the early culture period
				(ii) ECs increased *S. mutans* growth and virulent gene expression
				(iii) ECs increased adhesion and biofilm formation on teeth surfaces

Vemulapalli et al. 2021 [7]	In vivo observational (cross-sectional) study	Evaluated the link between vaping and untreated caries at the population level		(i) Higher levels of untreated caries in only EC smokers and dual smokers (EC + CCs)

Xu et al. 2022 [3]	In vitro	Evaluated the influence of flavors in e-liquids on single and multispecies biofilm formation, growth, and the inhibition mechanism	E-liquid flavor	Flavors:
				(i) Inhibit single-species and multispecies biofilms dose-dependently
				(ii) Produce bactericidal effect on the oral streptococci, inhibit oral commensal bacteria biofilm formation and growth, and negatively influence the oral microenvironment

Catala-Valentin et al. 2022 [20]	In vitro	Explored the influence of ECs on oral bacteria	EC aerosol	(i) EC aerosols disturb oral bacterial homeostasis by hindering commensal growth and promoting biofilm formation by the opportunistic pathogen *S. mutans*

## Data Availability

All the data used to support the findings of this review are included in the article.
